# Air-Coupled Piezoelectric Transducers with Active Polypropylene Foam Matching Layers

**DOI:** 10.3390/s130505996

**Published:** 2013-05-10

**Authors:** Tomás E. Gómez Álvarez-Arenas

**Affiliations:** UMEDIA Research Group, Centre for Physical Technologies, Spanish National Research Council (CSIC), Serrano 144, 28006 Madrid, Spain; E-Mail: tgomez@ia.cetef.csic.es; Tel.: +34-91-561-8806; Fax: +34-91-411-7651

**Keywords:** air-coupled ultrasound, air-coupled transducers, ferroelectret film, polypropylene piezoelectric foam

## Abstract

This work presents the design, construction and characterization of air-coupled piezoelectric transducers using 1–3 connectivity piezocomposite disks with a stack of matching layers being the outer one an active quarter wavelength layer made of polypropylene foam ferroelectret film. This kind of material has shown a stable piezoelectric response together with a very low acoustic impedance (<0.1 MRayl). These features make them a suitable candidate for the dual use or function proposed here: impedance matching layer and active material for air-coupled transduction. The transducer centre frequency is determined by the λ/4 resonance of the polypropylene foam ferroelectret film (0.35 MHz), then, the rest of the transducer components (piezocomposite disk and passive intermediate matching layers) are all tuned to this frequency. The transducer has been tested in several working modes including pulse-echo and pitch-catch as well as wide and narrow band excitation. The performance of the proposed novel transducer is compared with that of a conventional air-coupled transducers operating in a similar frequency range.

## 1. Introduction

Low frequency airborne ultrasound has been used for many years for applications like distance measurements, proximity sensors, defoaming and particle agglomeration. On the other hand, high frequency air-coupled transducers were initially intended to avoid the use of coupling liquids in fields like ultrasonic non-destructive testing and materials characterization. Nowadays, air-coupled ultrasonic transducers are finding new applications in different fields like contactless energy transfer (at 17 kHz using ECMT SA piezoelectric transducers [1,2]), wireless short range secure transmission of information (using 300 kHz, air-coupled micromachined capacitive transducers [3]) and human-machine interfaces, especially those related with touchless gesture recognition systems technologies (examples are the use of PZT, 40 kHz air-coupled transducers [4], 18–22 kHz speakers [5] and 100–500 kHz ferroelectret transducers [6]). These novel applications demand new transducer features and enhanced performance in some particular aspects. One possible research line to improve the performance and to increase the versatility of air-coupled piezoelectric transducers is the use of multiple piezoelectric layers as it has already been done in the past in other ultrasonic transduction fields (e.g., water coupled transducers for medical applications).

Initial works about ultrasonic transducers comprising multiple piezoelectric layers were mainly focused on techniques for bandwidth enhancement [7–9]. Hossack *et al.* proposed the use of synthetically generated waveforms to optimize net transducer performance [10,11]. Later, ultrasonic transducers having multiple active layers have been proposed for applications where bandwidth has to be enlarged or where two different operation frequencies or two different simultaneous modes of operation were required (e.g., therapy and imaging) [12,13]. The concept of active matching layers is then derived from the idea of a transducer having multiple active layers. In this case, one of these active layers is also used as matching layer. In this sense, Mulholland *et al.* [14] theoretically studied magnetically active materials in the matching layer so that, by applying an external magnetic field, the resonant behaviour of the device can be dynamically altered.

Application of this concept of active (piezoelectric) matching layer is promising in the context of air-coupled transducers. In this case, the impedance mismatch between the air and the piezoelectric ceramics or composites is very large and leads to poor sensitivities and narrow bandwidths. Use of impedance matching layers is compulsory in order to increase the sensitivity and enlarge the bandwidth; therefore, use of active matching layers can be a good option also in this field. The main problem is that the material requirements to produce impedance matching layers for air-coupled piezoelectric transducers are very demanding: very low acoustic impedances (0.01–0.1 MRayl) and low attenuation coefficient (<500 Np/m) are needed [15]. In general, it is not easy to find materials having these properties and, normally, they do not present any piezoelectric response. However, in the late 1980s a new kind of material was introduced that can meet some of these requirements: ferroelectrets.

Ferroelectrets are heterogeneous non-polar space-charge electrets that exhibit piezoelectric response combined with mechanical flexibility and low acoustic impedance (<0.1 MRayl) [16,17]. Ferroelectret films (thickness typically between 40 μm and 100 μm) are made of porous cellular polymers that contain elongated pores oriented within the film plane. Internal void surfaces can be charged by dielectric barrier discharges, so opposite charges are separated and trapped at the internal top and bottom surfaces of the voids [18,19]. A porous ferroelectret polypropylene (PP) film and fabrication procedure were patented by Kirjavainen in 1987 [20].

Ferroelectrets have been used to produce air-coupled ultrasonic transducers because their acoustic impedance is well matched to the impedance of the air [21–24], however, the piezoelectric response of these materials is reduced and the sensitivity of these transducers present no better performance compared with other solutions [25,26].

However, the very low acoustic impedance and the reduced thickness suggest the possibility to use them as conventional matching layers in the low MHz frequency range. Typical frequency location of the first thickness resonance of these films is between 0.3 and 0.6 MHz, so they can be used as active quarter wavelength (λ/4) matching layers in the low MHz frequency range (0.15–0.3 MHz).

The purpose of this paper is to present an air-coupled piezoelectric transducer with two active layers, one is a 1–3 connectivity piezocomposite made of a random distribution of piezoelectric fibres embedded in a polymeric matrix and the other one is a cellular polypropylene ferroelectret film. The design presents the special feature that the ferroelectret film can be used as both active layer (mainly as receiver) and as impedance matching layer (λ/4). The main ideas of this design are presented in Section 2. Towards this end, the first step is to characterize the ferroelectret film in order to determine its acoustic impedance and thickness resonant frequency (λ/4). It is also necessary to determine how the properties of this film are affected when it is attached to the rest of the transducer. The procedure proposed by Ealo *et al.* [23] has been followed. It consists on using an electrically conductive double sided adhesive tape (non-woven conductive scrim with acrylic adhesive, thickness 90 µm, density 1,100 kg/m^3^ and contact electrical resistance <0.4 ohm) to glue to the ferroelectret film to the substrate. The influence of this layer on the response of the ferroelectret film and on the whole transducers has also been considered. Then a 1–3 connectivity piezocomposite, whose first thickness resonance (λ/2) is tuned to the λ/4 thickness resonance of the ferroelectret, is selected. Two intermediate matching layers are then introduced so that the impedance profile along the transducer cross-section provides the optimum energy output. The thicknesses are calculated so that the frequencies of the λ/4 thickness resonances of these two layers are tuned to the resonant frequency of the ferroelectret (λ/4) and the piezocomposite (λ/2). Characterization of the main materials involved in the proposed transducer are shown in Section 3.

Then the performance of this transducer working in several different operation modes and under different excitation modes (spike and tone burst) is studied. Results are shown in Section 4. Finally, performance of this transducer is compared with the performance of a conventional air-coupled transducer constructed using a similar 1–3 piezoconnectivity composite, but without using active matching layers. This is shown in Section 5.

## 2. Design of the Transducer

**Figure 1.**
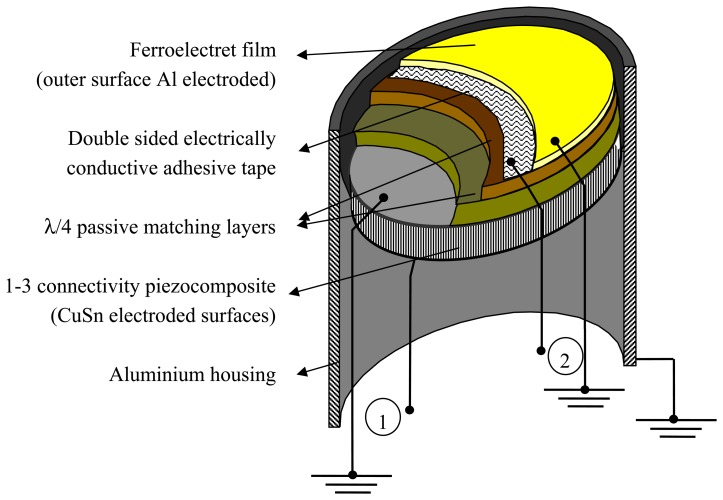
Schematic view of the cross-section of the air-coupled transducer with one active matching layer. 1: piezocomposite disk electrical terminals, 2: ferroelectret film electrical terminals. Ground connections are also indicated.

The cross section of the transducer design is shown in Figure 1. The outer layer is a ferroelectret film poled in the thickness direction and with the external face electroded with aluminium. It is attached to the rest of the transducer by using a double sided electrically conductive adhesive tape. Electrical connections are established with the electroded (outer) surface and to the electrically conductive adhesive tape so that it is possible either to apply an electrical voltage across the ferroelectret film (transmit mode) or to measure it (receive mode). Maximum energy output of the ferroelectret film is obtained at the thickness resonance that, for this design, takes place at the frequency determined by: λ/4, where λ is the wavelength. The transducer also contains a 1–3 connectivity piezoelectric composite, poled in the thickness direction and with electroded surfaces and electrical connections as before. In this case, maximum energy output is obtained at the thickness resonance that is located at the frequency determined by: λ/2. Therefore, the λ/2 resonant frequency of the piezocomposite and the λ/4 resonant frequency of the ferroelectret film must be tuned to the same frequency. Finally, there also are a couple of intermediate impedance matching layers between the ferroelectrect film and the piezocomposite.

**Figure 2.**
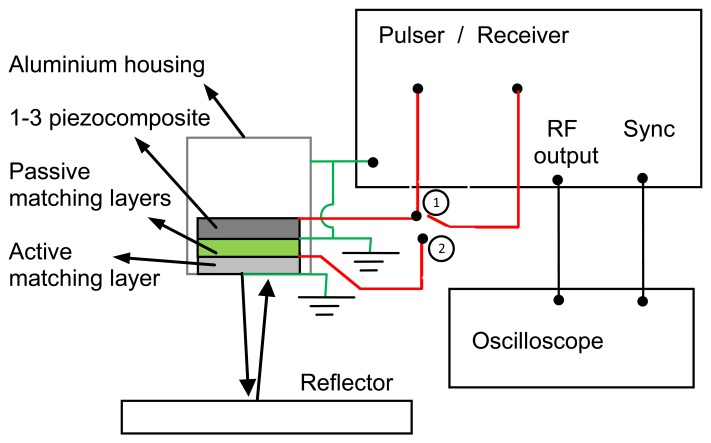
Schematic representation of some of the possible electrical connections of the air-coupled transducer with a piezoelectric matching layer. Switch is in position 1: transducer works in pulse-echo mode of operation (transmit and receive with the piezocomposite). Switch in position 2: transducer works in pitch-catch mode of operation: transmit with the piezocomposite and receive with the ferroelectret matching layer.

This design makes possible to operate the transducer in two different conventional pulse-echo modes. In one of them, the 1–3 connectivity piezocomposite is used as transmitter and receiver, while the rest of the layers (including the ferroelectret film) are used as conventional passive quarter wavelength matching layers. In the other mode, the ferroelectret film is used as both transmitter and receiver while the rest of the layers of the transducers only act as a mechanical loading. In addition, the transducer can be operated in pitch-catch mode in two different ways: (1) The piezocomposite acts as transmitter and the ferroelectret film as receiver and (2) The ferroelectret film acts as transmitter and the piezocomposite as receiver. Figure 2 shows a scheme of the electrical connections for two different cases of pulse-echo and pitch-catch modes of operation.

**Figure 3.**
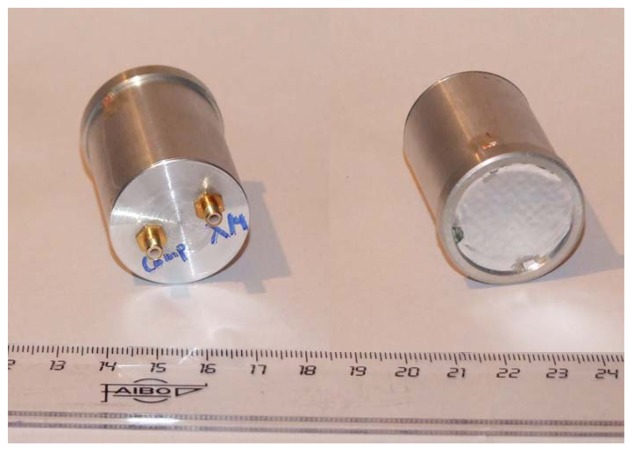
Pictures of rear (left) and front (right) faces of the prototypes of the proposed air-coupled transducer with active polypropylene foam matching layer.

Finally, both active layers can be operated simultaneously in order to improve the energy output of the transducer, increase the bandwidth or cancel the received/transmitted energy. Figure 3 shows some pictures of the prototypes.

## 3. Materials Properties

### 3.1. Ferroelectret Film

**Figure 4.**
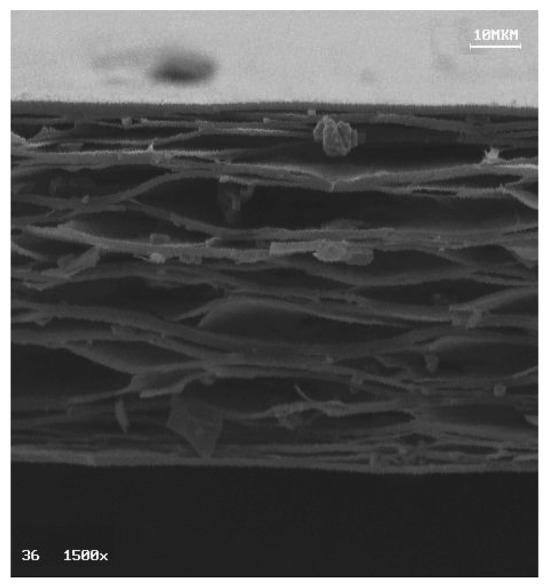
SEM image of the polypropylene ferroelectret film.

The used ferroelectret is a polypropylene (PP) cellular film, 70 μm thick, with a density of 540 kg/m^3^. One of the faces is electroded with an aluminium thin layer. A SEM image of the film cross-section revealing its cellular microstructure (high porosity and elongated pores) can be seen in Figure 4.

**Figure 5.**
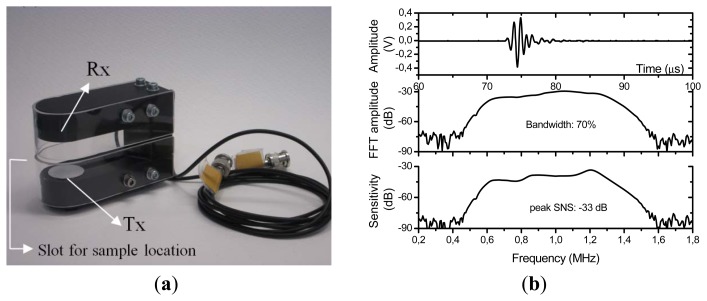
(**a**) Picture of the transmitter (Tx)-receiver (Rx) pair of transducers mounted on the system to perform through transmission measurements. (**b**) (Top) Impulse response of the Tx-Rx pair centered at 1.00 MHz. (Middle) FFT of the Impulse response. (Bottom) Sensitivity of the Tx-Rx system.

This PP film was first characterized by the analysis of its thickness resonances excited and sensed using air-coupled ultrasonic spectroscopy and a through transmission experimental set-up [27,28]. Several pairs of air-coupled transducers for these measurements were fabricated in our lab with centre frequency at 0.25, 1.00 and 2.00 MHz. They were mounted on a holder specially designed to perform through transmission measurements and for the characterization of solid plates by measuring the transmission coefficient at normal incidence. This holder allows to have the right transducers location, the right separation between them and also provides a slot for an easy location of the sample to be measured. Figure 5(a) shows a picture of the 1.00 MHz pair of transducers mounted on the measuring holder system. Figure 5(b) shows the impulse response of the 1 MHz pair of transducers (both in the time and the frequency domain -FFT-) and also the sensitivity, measured as the ratio of the FFT of the electrical signal measured in the receiver transducer to the FFT of the electrical signal measured in the receiver transducer terminals.

The ratio of incident to transmitted spectral wave amplitude (γ) can be calculated by solving the problem defined by the scalar wave potential amplitudes in each region of the space (air / film /air), the boundary conditions at the film/air interfaces, the constitutive equations for each material (foam film and air) and assuming plane wave and normal incidence [29]:

(1) $$\gamma =\frac{2}{2\sin\left(kt\right)+i\left(m+1/m\right)\sin\left(kt\right)}$$

where k is the wave number in the film (*k* = *ω*/*ν* +*i α*, where *ω* is the angular frequency, *ν* the ultrasound velocity and *α* the attenuation coefficient), *t* is the film thickness and *m* is the ration of film to air acoustic impedances.

**Figure 6.**
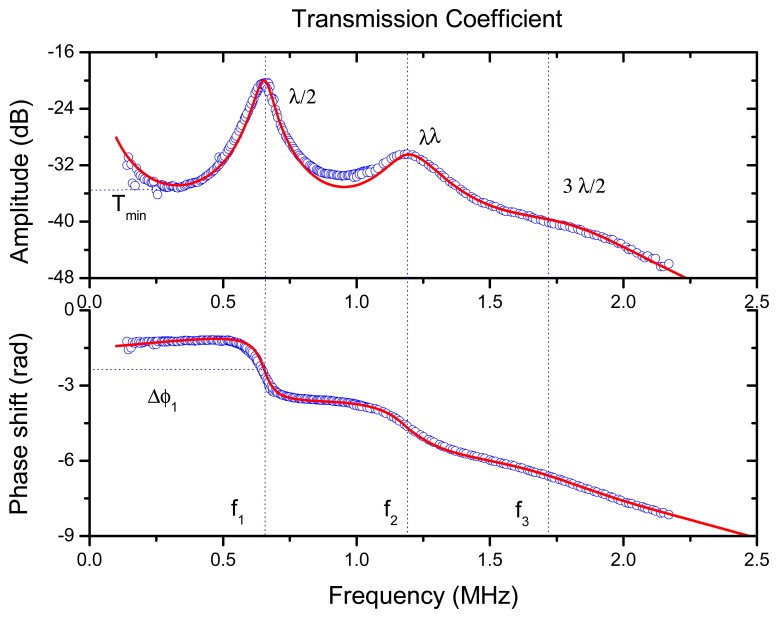
Spectra of the transmission coefficient of the PP film used for this work measured (blue circles) by air-coupled and wide-band ultrasonic transducers and calculated according to Equation (1): solid red line.

The measured transmission coefficient of the PP film is shown in Figure 6. It clearly reveals the first two orders of the λ/2 thickness resonances (located at 0.645 and 1.19 MHz). Using Equation (1) and experimental data in Figure 6 the inverse problem can be solve to extract the properties of the film: ultrasound velocity, density, impedance, attenuation coefficient and variation of the attenuation coefficient with the frequency (see [27]). Obtained value of the ultrasonic velocity (at 645 kHz) was 78 m/s, the acoustic impedance: 0.042 MRayl, the attenuation coefficient at the first order thickness resonance (645 kHz): 2,600 Np/m and the variation of the attenuation coefficient with the frequency follows a quadratic power law.

Though the value of the acoustic impedance is quite convenient to produce a quarter wavelength matching layer for air-coupled piezoelectric transducers, the attenuation is very high. A typical value of the attenuation coefficient at 650 kHz for other materials used for this application is between 100 and 500 Np/m. However, there are not too many options available to replace this material by any other with a lower attenuation and similar piezoelectric response. This is because attenuation is largely produced by the presence of the large pores which, on the other hand, are completely necessary to get a piezoelectric response.

**Figure 7.**
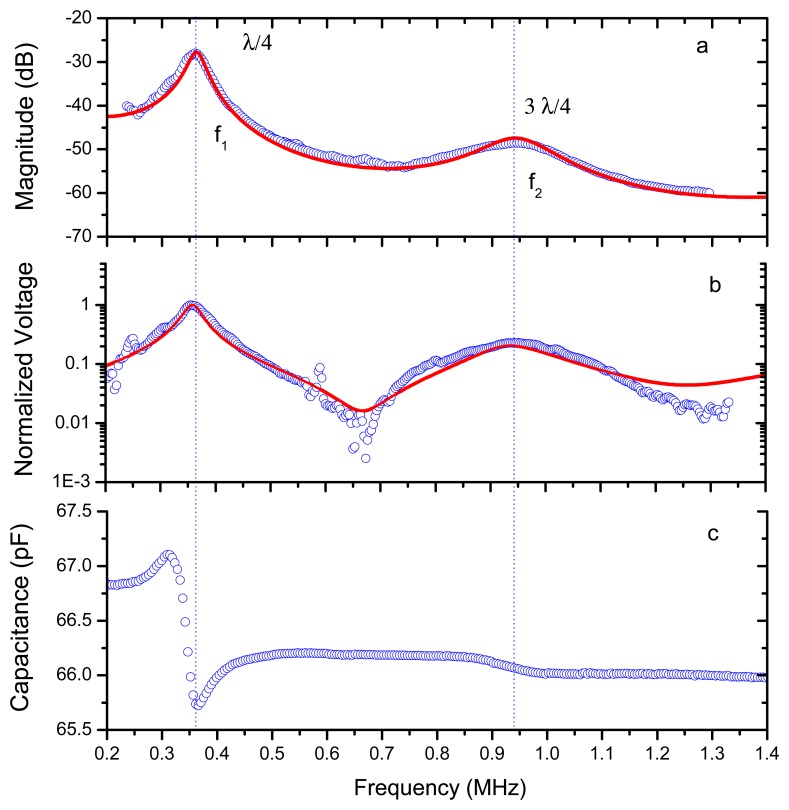
Measured (circles) and calculated (solid line) response *vs.* frequency of the PP film with a double adhesive electrically conductive layer glued to one surface. (**a**) Magnitude of transmission coefficient. (**b**) Normalized electrical voltage across the film generated by the incident acoustic beam. (**c**) Electrical capacitance.

It is of interest to determine the response of the λ/4 resonance of the PP film because this is the resonant mode that will be used in the final transducer design. We attached the film to the adhesive electrically conductive film used to glue the PP film to the transducer surface and to achieve an electrically conductive surface. Then this sample was characterized using the same technique as before: air-coupled ultrasonic spectroscopy and through transmission at normal incidence. Results are shown in Figure 7(a). As the acoustic impedance of this adhesive layer is very high compared with the acoustic impedance of the PP film, then the resonant frequency of the PP film is shifted from λ/2 to λ/4 (see Figures 6 and 7). As expected from measurements in Figure 6, the λ/4 resonance is located at 350 kHz.

In addition to the measurement of the transmission coefficient two other measurements were performed in this sample: (1) the voltage generated in the PP film as a result of the incident ultrasonic wave used for the through transmission measurements, and (2) the electrical capacitance. This latter measurement was performed by using a conventional impedance analyzer. These data can be used to determine the piezoelectric response of the ferroelectret film. All these measurements are shown in Figure 7.

The theoretical problem of sound transmission through a piezoelectric film is solved in [28], basically it is solved using the same procedure employed in the conventional problem of sound transmission through a wall (see [29] and Equation (1)), but now, the constitutive equations for the piezoelectric film are the constitutive equations of a piezoelectric material (see also [30]). The obtained analytical expression for the ratio of incident to transmitted spectral wave amplitude for one layer is now:

(2) $$\gamma =\frac{2r\Gamma }{2\Gamma \cos\left(kt\right)+i\left(1+r^{2}\Gamma^{2}\right)\sin\left(kt\right)}$$

where r is the ratio of wave vectors (air to film) and Γ = *λ*_1_/(*λ*_2_ + 2*μ*_2_), where λ and μ are the Lame's elastic constants of the air (1) and the PP film (2). The problem of a layered material can be solved with the same technique, but no analytical expression can be obtained. In this case, solution of the problem also allows us to obtain the electrical voltage across the PP-film terminals and the electrical impedance of any active layer.

### 3.2. 1–3 Connectivity Piezocomposite Disk

**Figure 8.**
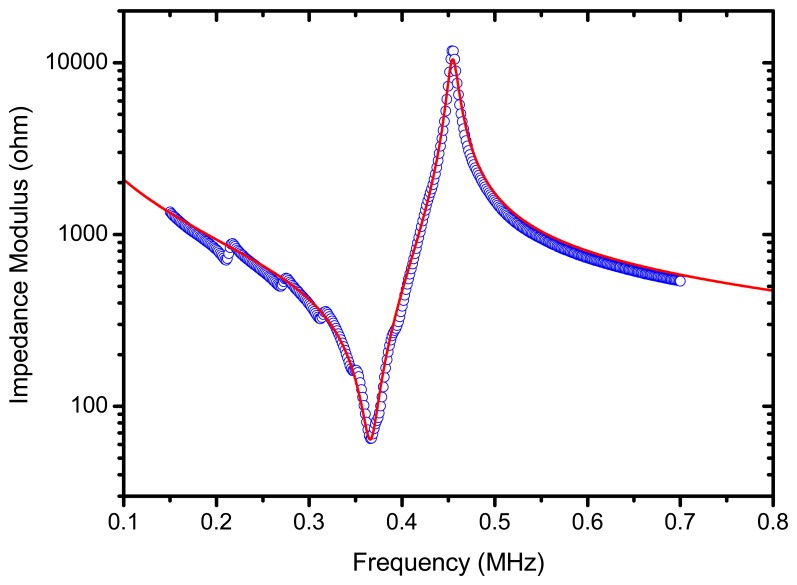
Modulus of the electrical impedance of the piezocomposite disk vs frequency. Circles: experimental measurements, solid red line: theoretical calculation.

The piezoelectric element is a random fibre 1–3 connectivity piezocomposite disk (ceramic volume fraction 65%) with acoustic impedance of 17 MRayl and diameter 25 mm, fabricated by Smart Materials Corp (Dresden, Germany). Fibres are oriented along the disk thickness direction. The disk is poled along the thickness direction and the surfaces are electroded (CuSn). A piezocomposite and not a piezoceramic was selected for three main reasons: (1) The acoustic impedance of the piezocomposite is lower; (2) The frequency bandwidth is larger, and (3) the influence of radial modes is almost negligible. The thickness of the piezocomposite disk is determined so that its first thickness resonance is located at 350 kHz, that is, the resonance of the piezocomposite is tuned to the resonance of the ferroelectret film. Figure 8 shows the measured electrical impedance of the piezocomposite disk *versus* frequency around the first thickness resonance. Radial modes appear only at low frequencies and are strongly attenuated, their influence on the thickness mode can be considered negligible. Theoretically calculated values are also shown. These calculations are performed assuming a one dimensional problem (see the expression for the electrical impedance of a piezoelectric disk poled in the thickness direction and with an electric field in the same direction [31]) and the following material parameters. Elastic constant in the thickness direction (*c_33_*): 72 GPa; density (ρ): 4,920 kg/m^3^; ultrasound attenuation coefficient at 0.35 MHz (α): 7 Np/m; relative dielectric constant at constant strain in the thickness direction (ε_33_/ε_0_): 360, and the piezoelectric constant in the thickness direction (*h_33_*): 3 × 10^9^; In addition, for the calculations it is assumed that the attenuation coefficient varies linearly with the frequency.

### 3.3. Additional Matching Layers

Between the piezocomposite disk and the ferroelectret film two intermediate quarter wavelength impedance matching layers are located. The role of these two layers is to enhance the sensitivity of the transducer and the bandwidth. Hence, the impedance of these matching layers (Z_L_) has been determined according to the criteria for maximum energy transmission between two media (Z_1_) and (Z_2_): Z_L_ = (Z_1_ + Z_2_)^1/2^. The first quarter wavelength matching layer is an epoxy resin layer (acoustic impedance 2.8 MRayl) tuned to 0.35 MHz. The attenuation coefficient of this material at 0.35 MHz is 3 Np/m. The second quarter wavelength matching layer is a porous polymer with a low acoustic impedance of 0.32 MRayl [15] and attenuation coefficient at 0.35 MHz of 550 Np/m.

### 3.4. Summary of Materials Properties

Main materials properties are summarized in Table 1.

## 4. Transducer Characterization

### 4.1. Electrical Response of the Transducer

**Figure 9.**
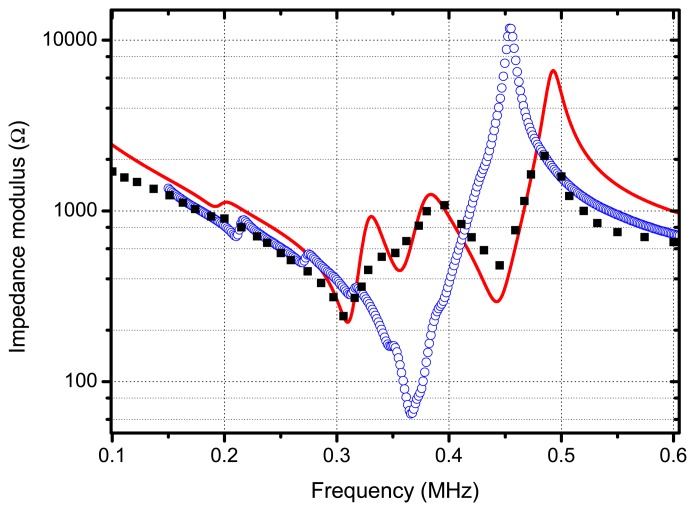
Electrical impedance (modulus) *versus* frequency of the final transducer measured in the terms of the piezocomposite disk. Black dots measurements, solid red line: calculations. Open circles: measured impedance of the piezocomposite disk.

The electrical impedance of the transducer (measured across the piezocomposite disk terminals) is shown in Figure 9, in addition, the calculated values assuming a multilayered 1-D model for the transducer (see [30]) and using the materials data before mentioned, are also shown. For comparison purposes the electrical impedance of the piezocomposite disk alone (*i.e.*, without any matching layer attached) is also displayed. Both the increase of the minimum value of Z and the decrease of the maximum are due to the effect of the mechanical load of the matching layers on the piezocomposite disk. The appearance of new relative maxima and minima is due to the resonant behaviour of the matching layers and to the fact that these resonances are all tuned to the electrical resonance of the piezocomposite disk.

### 4.2. Pulse-Echo Transducer Response under Broad-Band Excitation (Spike)

**Figure 10.**
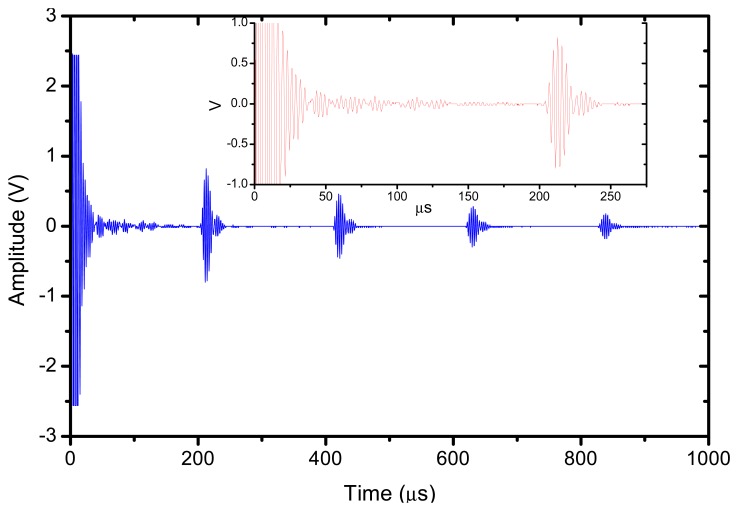
Transducer response in pulse-echo with the 1–3 connectivity piezocomposite layer.

**Figure 11.**
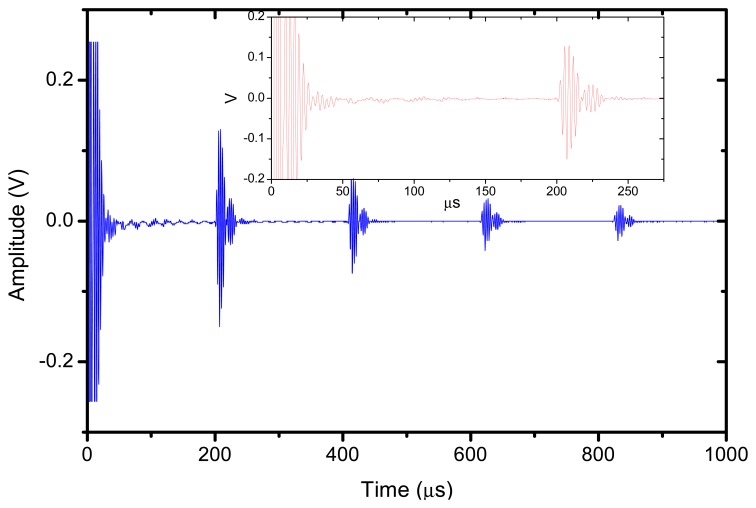
Pitch-catch response. Transmitter: 1–3 connectivity piezocomposite, Receiver: ferroelectret film.

**Figure 12.**
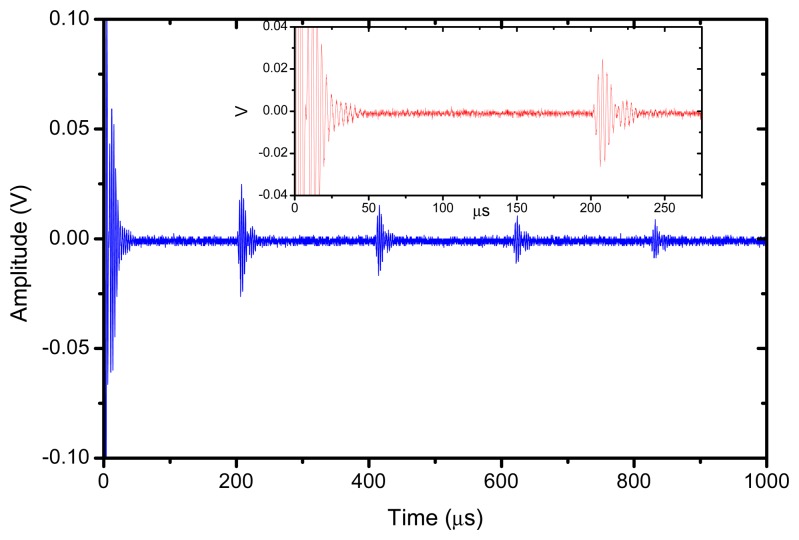
Pulse-Echo (transmit and receive) with the ferroelectret film.

**Figure 13.**
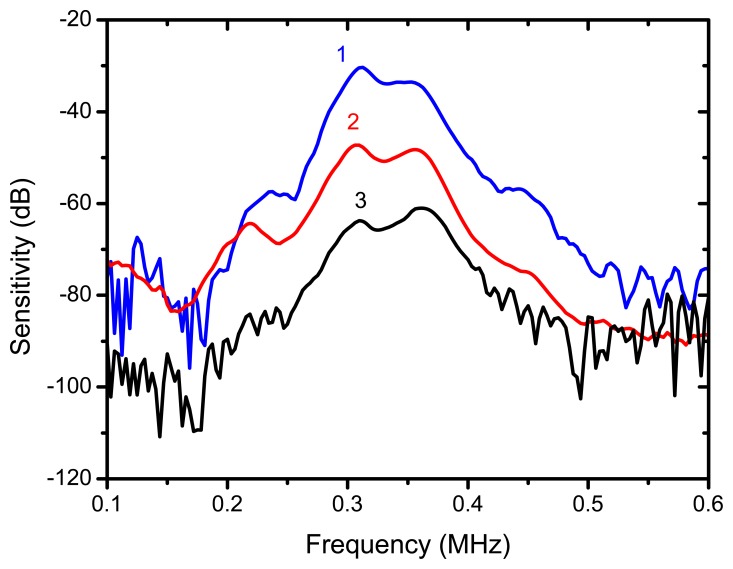
Sensitivity vs frequency for three different operation modes of the transducer. 1: (Blue) Pulse-echo with the 1–3 connectivity composite (6dB-bandwidth 25%). 2: (Red) Pitch-catch, transmit with the 1–3 connectivity piezocomposite and receive with the ferroelectret film (6 dB bandwidth 28%). 3: (Black) Pulse-echo with the ferroelectret film (6-dB bandwidth: 28%).

The reflector was a steel block located at 36 mm from the transducer surface. The pulser receiver (P/R) was a Panametrics 5077 and the oscilloscope a Tektronix DPO5052. The P/R drives the transmitter with a spike, 200V amplitude. The amplification in reception is set to 0 dB. No analog filtering was used in reception. Figures 10, 11 and 12 show the received signal displayed in the digital scope when the transducer operates in three different modes: (1). Pulse-echo with the 1–3 connectivity composite; (2). Pitch-catch: transmit with the 1–3 connectivity piezocomposite and receive with the ferroelectret film; (3). Pulse-echo with the ferroelectret film. The shape of the received pulse is similar for all cases, while the amplitude presents significant variations. Figure 13 shows the sensitivity (defined as the ratio of measured voltage in reception to the applied voltage across the terminals of the active layer used as transmitter, see Equation (3) for each of these configurations:

(3) $$S=20\log_{10}\left(\frac{V_{\text{rec}}}{V_{0}}\right)$$

The highest sensitivity is obtained when the piezocomposite disk acts as transmitter and receiver, that is, the ferroelectret film is used a conventional passive matching layer. On the other hand, when the ferroelectret film is used as both transmitter and receiver the sensitivity is about 30 dB below. Bandwidth is similar for the three cases.

### 4.3. Pulse-Echo Transducer Response under Narrow-Band Excitation (Tone Burst)

**Figure 14.**
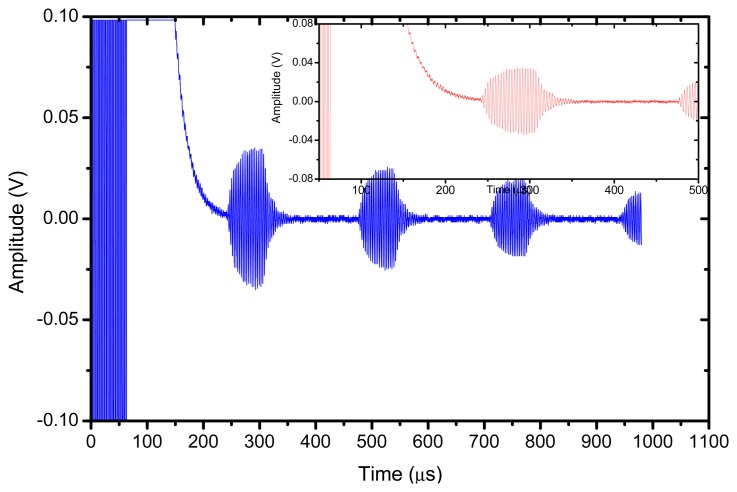
Pulse-Echo with the piezocomposite disk. Tone burst excitation at 315 kHz.

**Figure 15.**
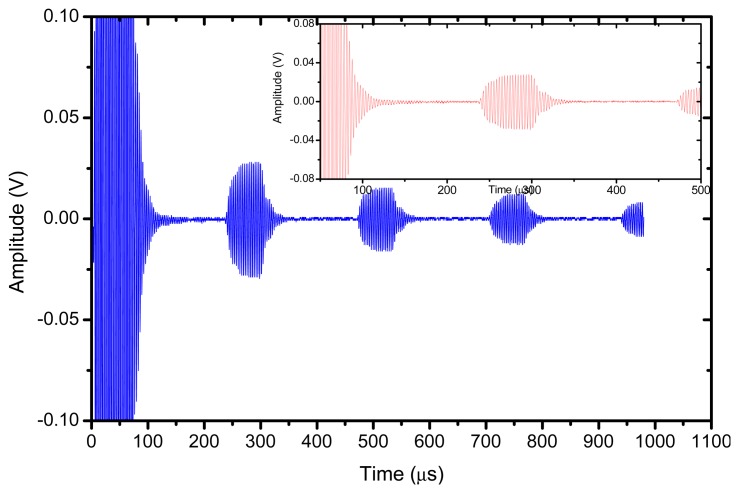
Pitch-Catch. Transmitter: piezocomposite disk, receiver: ferroelectret film. 315 kHz.

The response of the transducer is also studied under narrowband excitation. An Agilent arbitrary function generator was used. A 10 V amplitude and 20 cycles sine wave was used to drive the transducer. The steel block reflector was located at 36 mm from the transducer surface. The electrical signal applied across the transducer terminals is directly measured and displayed by the oscilloscope, results are shown in Figures 14 and 15. In this case, the largest amplitude of the received signal is obtained for the pulse-echo mode when the piezocomposite disk acts as transmitter and receiver, the pitch-catch mode (with the piezocomposite disk as transmitter and the ferroelectret film as receiver) presents a very similar sensitivity. In addition, the pitch-catch mode (transmitter: piezocomposite, receiver: ferroelectret) present the additional advantage compared with the pulse-echo mode that the effect of the electrical excitation is reduced and it is possible to measure the echo of an object more closely located to the transducer surface.

## 5. Comparison with a Conventional Air-coupled Transducer

**Figure 16.**
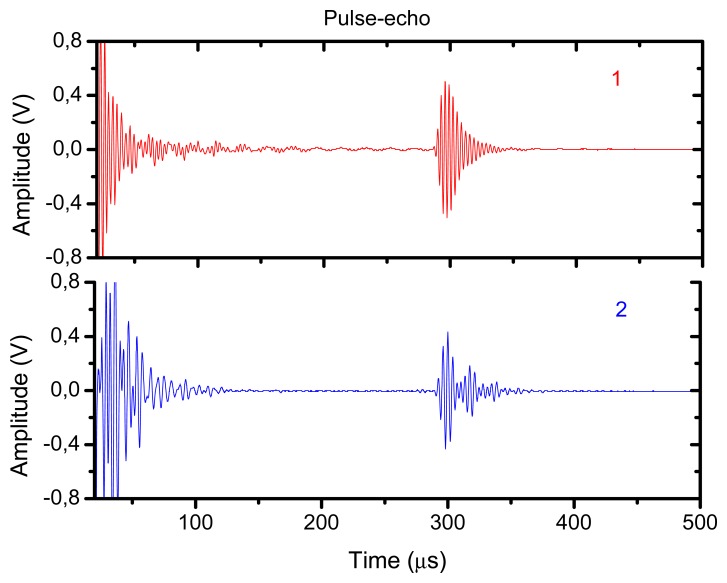
Pulse-echo mode of operation, wideband excitation (spike). 1. (Red) Air-coupled transducer with active matching layer. 2. (Blue) Conventional air-coupled transducer with passive matching layers.

**Figure 17.**
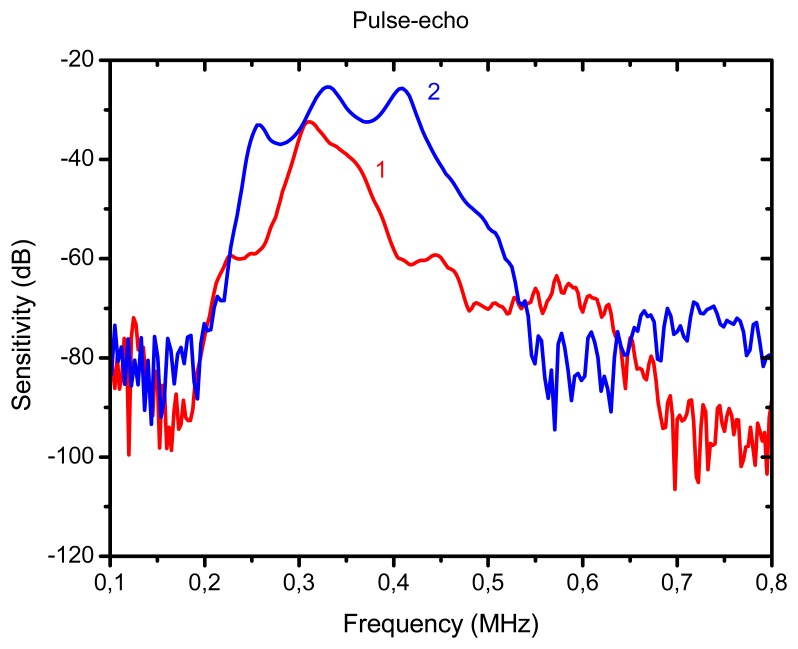
Sensitivity in pulse-echo mode of operation wideband excitation (spike). 1. (Red) Air-coupled transducer with active matching layer. 2. (Blue) Conventional air-coupled transducer with passive matching layers.

**Figure 18.**
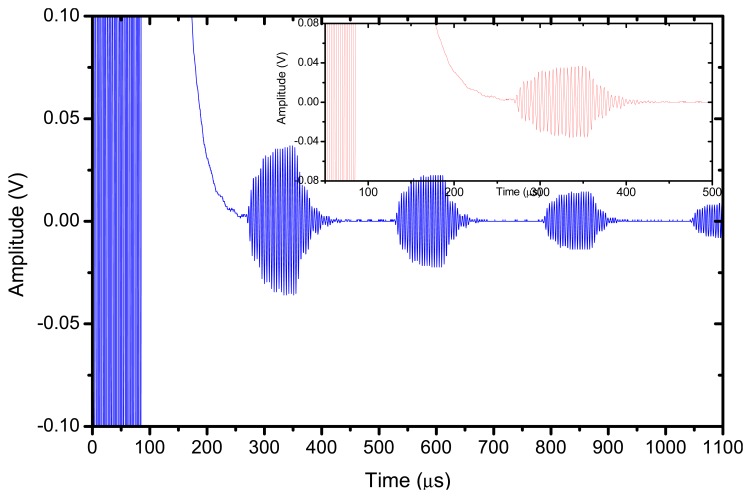
Pulse-Echo mode of operation, narrow band excitation (tone burst). Conventional air-coupled transducer with passive matching layers (compare with Figures 13 and 14). Reflector at 45 mm.

For comparison purposes we also show the response of a conventional air-coupled transducer [10] operated under similar conditions. The purpose is to determine to what extent the excess of attenuation in the active matching layer and the presence of an electrically conductive layer limit the performance of the proposed new transducer in comparison with a transducer that does not present these problems. The same piezoelectric element (piezocomposite ceramic disk, diameter 25 mm), and the same housing, as before, were used to produce these transducers, so the unique difference is on the different matching scheme employed. In this case, three conventional and passive λ/4 impedance matching layers were used (impedances of 2.5, 0.7 and 0.074 MRayl and attenuation coefficient at resonance 12, 120 and 500 Np/m). It can be appreciated that the attenuation in the outer matching layer in this case is much lower than the attenuation in the ferroelectrect foam film used as the active matching layer. The wideband pulse-echo response is shown in Figures 16 and 17. The peak sensitivity in pulse-echo operation is a little bit better for the conventional transducer (−25 dB) and the useful bandwidth is larger, however, the performance of the transducer with the active matching layer is not far behind. This latter transducer does have the advantage of permitting different modes of operation. Figure 18 shows the pulse-echo response for narrow band (tone burst) excitation.

## 6. Conclusions

This work presents a novel design for air-coupled transducers with one active quarter wavelength matching layer made of a polypropylene ferroelectret film. The main idea of the presented design is to exploit the low impedance value of ferroelectret films to use them as both conventional quarter wavelength matching layer and also as an active layer. Then the transducers can be operated either in the conventional pulse-echo mode (using the same active layer to transmit and to receive) or in a dual pitch-catch mode (where different active layers are use to transmit and receive).

First, the ferroelectret films were characterized and the attenuation coefficient, acoustic impedance and frequency location of the λ/2 resonance were determined. The main drawbacks of this material are the large attenuation coefficient and the lower sensitivity (when compared with piezocomposites). Then, the ferroelectret λ/4 resonance (the response of the film once attached to a substrate) was studied. The rest of the transducer layers (1–3 piezocomposite active layer and passive matching layers) were all tuned to this λ/4 resonance.

This transducer can be operated in conventional pulse-echo mode, with a performance similar to other conventional air-coupled transducers, or in a dual mode (transmit with the piezocomposite and receive with the active matching layer). In this case, sensitivity is reduced but other advantages, as the reduction of the transducer dead zone for pulse-echo operations, can be achieved.

Other uses of this technology such as the use of the active matching layer to enhance the emission or the simultaneous use of both received signals to improve the resolution will be investigated in the future.

## Figures and Tables

**Table 1.** Summary of materials properties.

**Material**	**Description**	**Impedance (MRayl)**	**Resonant frequency (kHz)**/**(Type of resonance)**	**Attenuation at resonant frequency**
**Piezocomposite**	1-3 connectivity composite (random fibers)	17	350 (λ/2)	7
**Electrically conductive adhesive tape**	non-woven conductive scrim with acrylic adhesive	1.2	About 10 MHz (λ/2)	∼750
**First matching layer**	Epoxy resin	2.8	350 (λ/4)	3
**Second matching layer**	Porous polymeric film [15]	0.32	350 (λ/4)	550
**Active outer matching layer**	Polypropylene ferroelectret film	0.042	340 (λ/4)	650
